# A Rapid Review and Narrative Synthesis of the Consequences of Non-Inclusive Sex Education in UK Schools on Lesbian, Gay, Bisexual, Transgender and Questioning Young People

**DOI:** 10.1177/10598405211043394

**Published:** 2021-10-04

**Authors:** Beth Epps, Marianne Markowski, Karen Cleaver

**Affiliations:** 1 Kent School Health Team, Children and Young People's Directorate, Kent Community Health NHS Foundation Trust, Maidstone, UK; 2 Institute for Lifecourse Development, Faculty of Education, Health and Human Sciences, 4918 University of Greenwich, London, UK; 3 Faculty of Education, Health and Human Sciences, 4918 University of Greenwich, London, UK

**Keywords:** inclusive RSE, relationship and sex education, health education, middle/junior/high school, lesbian, gay, bisexual transgender, questioning

## Abstract

Relationships and Sex Education (RSE) in schools are predominantly heterocentric. Consequently, lesbian, gay, bisexual, transgender, and questioning young people have reported feeling excluded. This exclusion results in feelings of being “different” and “other,” which in turn leads to further disengagement in the sex education classroom, contributing to poor sexual health literacy, greater risk of abusive relationships, and higher rates of sexually transmitted infections. A rapid review was undertaken to identify the impact of non-inclusive sex education. The review makes recommendations for policy and practice, which includes the provision of training courses to school teaching staff with an emphasis on inclusive RSE, appropriate online resources for lesbian, gay, bisexual transgender, and questioning (LGBTQ) young people, as well as offering 1:1 emotional health support for LGBTQ young people as they begin to question their sexual orientation.

## Introduction

Young people in the United Kingdom (UK) who identify as lesbian, gay, bisexual transgender, and questioning (LGBTQ) are more likely to develop anxiety, low self-esteem, depression, self-harm, and substance misuse (Metro, 2016; ONS, 2017; Stonewall, 2017), a consequence of homophobia, biphobia, transphobia, social exclusion and rejection by peers and family (Mind, 2020). From a sexual health perspective, LGBTQ youth are more likely to engage in sex with high numbers of partners and first experiences of sex are more likely to be under the influence of alcohol (Metro, 2016), exposing these young people to greater risk of sexually transmitted infections (STIs). In 2018, 47% of Gonorrhea and 75% of syphilis diagnoses in the UK were in men who have sex with men (Public Health England, 2019), which can be linked to both higher numbers of sexual partners as well as reduced levels of health literacy, a result of disenfranchisement in the sex education classroom (Aranda et al., 2018; Coll et al., 2018; Estes, 2017; Formby & Donovan, 2020; Gowen & Winges-Yanez, 2014; Grant & Nash, 2018; Hobaica & Kwon, 2017; Hoefer & Hoefer, 2017; Keiser et al., 2019; Snapp et al., 2015). Inclusive sex education is, therefore, an imperative to improve both the emotional and sexual health of LGBTQ young people. This paper reports on a rapid review, which aims to provide a synthesis of current knowledge of how current approaches to school-based sex education influence the sexual health and wellbeing of young people who identify as LGBTQ.

### Background

Relationship and sex education (RSE) is built on the understanding that “real sex” is penis to vagina, meaning that anything else is out of the ordinary and alternative (Grant & Nash, 2018; Hoefer & Hoefer, 2017; Moran, 2001), thereby alienating young people who identify as LGBTQ (Abbott et al., 2015; Gowen & Winges-Yanez, 2014). This disengagement creates poor sexual health literacy and less safe sexual practice (Estes, 2017; Grant & Nash, 2018; Hoefer & Hoefer, 2017) A Stonewall survey of LGBTQ young people in the UK found that although 60%–80% had been taught about contraception, safe sex, violence in relationships and consent, only 20% had been taught about these issues from a same-sex partners perspective (Stonewall, 2017). This lack of formal RSE relevant to LGBTQ youth causes young people to source this information through their early sexual partners, which leaves them at risk of sexual exploitation and violence within their relationships (Burkill & Waterhouse, 2019; Formby & Donovan, 2020; Hobaica & Kwon, 2017; UNESCO, 2018).

RSE has the capacity to educate young people and promote good sexual health decision-making. Indeed, comprehensive sex education programs have demonstrated the ability to decrease teen pregnancy and delay initiation into sex (Rabbitte & Enriquez, 2019). However, RSE is traditionally delivered from a heterocentric standpoint (Elia & Eliason, 2010). Due to a climate of homophobia in schools (Metro, 2016; Stonewall, 2017), many LGBTQ young people struggle to voice their opinion on the sex education they receive, which perpetuates the standard heterocentric approach to RSE. This is important as inclusive sex education postpones the onset of sexual intercourse for all young people irrespective of their sexual identity (Chin et al., 2012).

In recognition of the changing context of young peoples’ lives, notably with widespread access to the internet and the resultant potential for grooming and child sexual exploitation, the UK introduced legislation making RSE compulsory for all UK children and young people aged 11 and over (DfE, 2019, p. 45). Government guidance related to RSE was subsequently updated (DfE, 2019) and now requires schools to ensure that students understand the characteristics of a healthy relationship, including same-sex relationships. However, talking to young people about sex can be discomfiting, particularly in the context of same-sex relationships, as some view discussing same-sex relationships with young people as promoting immoral or risky sexual experimentation (Attwood & Smith, 2014; Grant & Nash, 2018).

School nurses representing the UK school health service can provide guidance to schools on how to teach RSE. For this to occur, school nurses need to stay abreast with the latest regulative developments and consult or refer to school-based health centers. A study by Garbers and colleagues found there are significant gaps and strong regional differences in the extent to which school health services provide culturally competent care for young people who identify as LGBTQ (Garbers et al., 2018). It is important, therefore, to understand how these young people experience their school-based RSE. This review, therefore, aims to identify existing research, which examines the experiences of young people who identify as LGBTQ in relation to school-based RSE.

## Method

### Design

A rapid review was undertaken. A rapid review provides a mapping of available research enabling a rapid synthesis of knowledge about a policy or clinical practice issue and attempts to inform an evidence-based decision (Khangura et al., 2012; Tricco et al., 2015). The principles of a rapid review are outlined below in Table 1.

**Table 1.** Principles of Rapid Review Methodology (Khangura et al., 2012, p2).

Timeframe	≤ 5 weeks
Question	Question specified *a priori* (may include broad PICO, which stands for Population, Intervention, Comparison and Outcome)
Sources and searches	Sources may be limited but sources/strategies made explicit
Selection	Criterion-based; uniformly applied
Appraisal	Rigorous; critical appraisal
Synthesis	Descriptive summary/categorization of the data
Inferences	Limited/cautious interpretation of the findings

The review was conducted according to the Preferred Reporting Items for Systemic Review and Meta-Analysis guidelines (Moher et al., 2009). The authors applied the approach of narrative synthesis to present the results (Mays et al., 2005a; Mays et al., 2005b).

The guiding research questions were:

1. What are the reported experiences of LGBTQ young people receiving school-based RSE?
2. How does the approach to RSE adopted affect LGBTQ young peoples’ emotional and sexual health?

### Search Methods

The search took place in April 2020 using EBSCOhost, providing access to Academic Search Premier, MEDLINE, Psychology and Behavioral Sciences Collection, PsycInfo, and CINAHL databases. The following search terms were used: “school sex and relationships education*” AND “LGBTQ* OR lesbian* OR gay OR homosexual* OR bisexual* OR transgender* OR homosexual* OR queer* OR sexual minority*.” The database parameters were set to include only peer reviewed articles, published in English from 2010.

### Inclusion/Exclusion Criteria

The initial search returned 440 papers, reduced to 275 following the removal of duplicates.

Inclusion criteria were:

- Empirical/primary peer reviewed research articles
- Quantitative, qualitative, and mixed method studies
- Studies that directly involve the views of LGBTQ young people who had accessed school-based RSE

Exclusion criteria were:

- The research was carried out in a country with a non-comparable culture/education system
- Conference papers, commentary, opinion pieces

**Figure 1.**
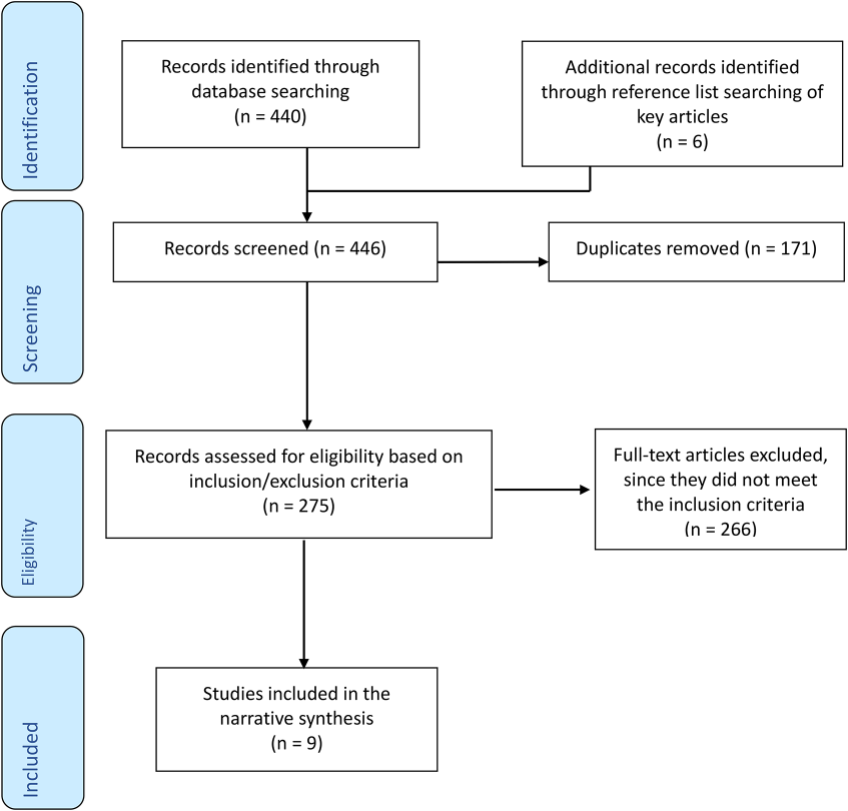
Preferred reporting items for systemic review and meta-analysis (PRISMA) diagram.

A total of 266 articles did not meet the inclusion criteria; the nine papers which met the criteria were subject to quality review and were then included in the qualitative synthesis (see Figure 1).

### Data Extraction

Data extraction was undertaken by the first author. Data were categorized according to country, year of publication, study aims, sample characteristics, research design, and data collection, and main findings (see Table 2).

**Table 2.** Extraction Table.

Author	Country and year of publication	Study aims and objectives	Sample size	Research design and data collection	Main findings
Baams et al. (2017)	2017 The Netherlands	Longitudinal study to explore whether the whether the specific content of sexuality education or the extensiveness of sexuality education is related to an improvement of school climate over time	Sample after the third wave: (*N* = 577) adolescent from six different schools	Three wave survey to measure and track responses on (1) Sexuality education and (2) The occurrence of LGBTQ name-calling and perceived willingness to intervene	Having more extensive sexuality education, and having more topics covered, was related to an increase in the willingness to intervene when witnessing LGBTQ name-calling. Male and female adolescence differed in the perceived willingness to intervene. Females reported a higher tendency to intervene when witnessing LGBTQ name-calling than males (ps < .05).
Coll et al. (2018)	2018 Ireland, UK	Examined young peoples views on what sexuality education ought to be about.	*n* = 27 Year 1 *n* = 16 Year 2	Youth participatory Action Study Focus Groups and semi-structured interviews.	Approaches to sex education are entrenched and do not listen or respond to the views of young people and the information they need to know.
Estes (2017)	2017, North America, USA	Examined how LGBTQ young people understand their experiences of sex education in the home, at school, and from outside sources.	LGB young adults aged 19–25. (*n* = 10)	Qualitative. Semi-structured interviews were analysed using grounded theory	RSE is heterosexually centered; revolves around sex as being “dangerous” and is often lacking the most basic health and behavior information. Parents base their discussions on heterosexual relationship discussions even if they are aware of their child's sexuality. The young adults obtained sexual information mostly through the internet or LGB-specific TV programs, where not all information was accurate, educational, or realistic.
Formby and Donovan (2020)	2020, UK	Examined the rationale for improving sex education to support young people's mental health and practitioners' experiences of delivering RSE	Young people aged between 14-24: focus group (*n* = 7) & descriptive surveys (*N* = 119) Interviews with staff (*n* = 6)	Qualitative. Focus groups, descriptive surveys, and interviews	LGBTQ young people felt left out of sex education—had to self-educate, leading to unsafe relationships and sexual encounters. Participants of this study wanted more education on same-sex relationships in relation to domestic abuse.
Gowen and Winges-Yanez (2014)	2014, North America	Examined the perspectives of LGBTQ youth on their experiences with school-based sexuality education in order to create a framework of LGBTQ-inclusive sexuality education	(*n* = 30) aged between 16-20	Qualitative. 5 × Focus groups and thematic analysis	Silencing; actively or passively by closing down discussions of sexuality. Heterocentricity; curriculum based on assumption that all students are heterosexual. Participants felt their sexuality was pathologized.
Grant and Nash (2018)	2018, Australia	Explored the understanding of bisexual and queer young women of “safe sex” and how they conceptualize “good” sexual citizenship in rural Australia.	lesbian and bisexual young women aged 19–26 (*n* = 15)	Qualitative Semi-structured interviews analysed using grounded theory and informed by feminist research principles	Tasmanian LBQ young women experience an absence of safer sexual scripts and decreased sexual health literacy. Good sexual citizenship is established as heteronormative. Participants disengaged due to heterocentric sex education. Sex as a risk is too heavily relied upon by educators.
Hobaica and Kwon (2017)	2018, North America	Examined sexual minorities' experiences of their sex education	sexual minorities aged 19-38 years (*n* = 12)	Qualitative. Semi-structured interviews were analysed using grounded theory	Sex education explained how to be heterosexual with no information on how to stay safe during other types of sex (oral/anal); Feelings of fear about having sex; not enough info on pleasure, positive emotions, or consent. Only half of the sexual experiences were safe with evidence of non-consensual sex. Mental health concerns resulting from issues “hiding part of yourself”/internalized homophobia.
Hoefer and Hoefer (2017)	2017, North America (Texas)	Examined the impact of abstinence only sexual education on marginalized groups.	(*N* = 16) students (recruited as female, LGBTQ or person of color) from across the state	Qualitative. Semi-structured interviews using a feminist framework for analysis	Lack of information and resources; inclusion of sexist and heterosexist stereotypes; teacher adultification of a young person of color; lack of emotional safety and need to hide; teachers' reliance on fear and shame.
Keiser et al. (2019)	2019, North America	To explore the aspects most associated with the perception of inclusion in sex education among sexual minorities, to develop a measure to assess these aspects of sex education perceptions, and to investigate the mental and sexual health outcomes associated with perceived inclusivity and exclusivity in sex education curricula.	Young adults aged 18–26 years (*n* = 263)	Survey using the Perceived Inclusivity of Sex Education Scale	Greater perceived inclusivity was associated with lower anxiety, depression, and suicidality for sexual minorities.

LBQ = lesbian bisexual queer; LGB = lesbian gay bisexual; LGBTQ = lesbian, gay, bisexual, transgender and questioning; RSE = relationships and sex education; PISES = perceived inclusivity of sex education scale.

### Data Synthesis

As the narrative synthesis is predominately a “framework” to present the results (Mays et al., 2005b, p. 6), authors employ methods of their choice (e.g. thematic analysis, meta-ethnography, qualitative cross-case analysis) in order to work with the data. We decided to employ thematic analysis (Braun & Clarke, 2006) adopting both an inductive and deductive approach. The first author reviewed the literature and extracted initial themes around the experience, context, and consequences for LGBTQ youth, and grouped the articles by the focus of the research. The second and third authors reviewed the initial groupings and labeling of the themes and repeated this process from thereon. After negotiation between all three authors, we arrived at the final three themes to present the synthesis.

## Results

### Quality Appraisal Results

The nine articles were critically appraised using the Critical Appraisal Skills Checklists (CASP). Overall, the results of the critical appraisal showed that the articles presented were good to medium quality research. For seven articles the qualitative checklist (CASP, 2019a) was utilized; three articles scored “yes” in all questions (Coll et al., 2018; Gowen & Winges-Yanez, 2014; Grant & Nash, 2018); five articles received a “Can’t tell” for the question whether the relationship between researcher and participants has been adequately considered, with one of those articles receiving a further “No” regarding recruitment (Estes, 2017), and one receiving two “no’s for research design,” recruitment and one “can’t tell” for a clear statement of findings (Hoefer & Hoefer, 2017). Two articles (Baams et al., 2017; Keiser et al., 2019) was assessed using the CASP cohort study checklist (CASP, 2019b) with both receiving a “Can’t tell” for the accuracy of exposure to minimize bias, and Keiser et al. (2019) also received “Can’t tell” for completeness and length of the follow-up of subjects.

### Descriptive Summary of the Results

All studies were conducted between 2014 and 2019 and involve a total sample of 1,085 young adults as participants. Five studies were conducted in North America (Estes, 2017; Gowen & Winges-Yanez, 2014; Hobaica & Kwon, 2017; Hoefer & Hoefer, 2017; Keiser et al., 2019) one from Australia (Grant & Nash, 2018), one from the UK (Formby & Donovan, 2020), one from Ireland (Coll et al., 2018) and one from the Netherlands (Baams et al., 2017). Of the nine articles, six studies included participants who classified themselves as lesbian, gay, transgender, or questioning, which accrues to a combined sample of 202 participants who identified as sexual minorities. One article featured only self-identified queer young women (Grant & Nash, 2018).

All qualitative articles used purposive sampling to select LGBTQ young people in order to speak to participants who had experienced the phenomenon of heterocentric sex education from the viewpoint of a sexual minority (Ellis, 2016). One paper (Baams et al., 2017), reported that only the question around the biological sex was a mandatory in the survey and therefore not all minorities were captured in the data, but the paper was still included since questions focused on sexuality education in particular. All articles adhered to ethical considerations considering the age of their participants, although consent was not always gained from parents of young people under the age of 18 years due to confidentiality around the young person’s sexual identity.

Seven of the nine studies employed qualitative methodologies, predominantly interviews and focus groups. Grounded theory was utilized in three articles to provide an open account of the ideas emerging from each study (Estes, 2017; Grant & Nash, 2018; Hobaica & Kwon, 2017). One article (Coll et al., 2018) employed a Youth Participatory Action Research method, which is a model used to engage youth and create transformational change within communities with young people involved as active researchers. Another article (Formby & Donovan, 2020) portrayed the collaboration between an arts organization and two universities, where participants revealed their experiences and needs while creating arts & crafts to express themselves.

### Narrative Synthesis Results

Three themes were developed for the narrative synthesis and guide the presentation of the results. Firstly, students feel schools are heterocentric in their approach to sex education; secondly, LGBTQ young people feel that they were left unprepared for their relationships and sexual lives the education they received perceived as being irrelevant to them; and thirdly in lieu of relevant school-based sex education, LGBTQ young people access the internet to educate themselves on their sexual orientation.

#### Theme 1: Heterocentric Schools

This theme presents a synthesis of findings, which illustrate that not only is RSE pre dominantly heterocentric, but the schools also are heterocentric environments. Moreover, without inclusive RSE schools are likely to remain heterocentric, thus unintentionally facilitating the bullying of sexual minorities. Heterocentric RSE has an evident impact on emotional health, and the self-esteem and identity of LGBTQ youth, whose needs are neither addressed nor included.

The participants in Gowen and Winges-Yanez's (2014) qualitative study reported that as RSE focused only on penis to vagina sexual intercourse and pregnancy prevention, many young people disengaged and did not pay attention, leading to feelings of isolation and disengagement. Likewise, Hobaica and Kwon (2017) conducted interviews with 12 sexual minority participants aged between 19 and 38 years. Findings from this study demonstrate that all students received limited information on anything other than heterosexual sex and relationship education, leaving them feeling unsure how to identify as a sexual minority and begin healthy relationships. One participant noted that she *“couldn't relate to anybody or anything they were talking about”* (Hobaica & Kwon, 2017, p. 12). Lack of discussion around pleasure in sex also caused the participants in Hobaica and Kwon's (2017) study to engage in heterosexual relationships, as they were not aware that sex should be pleasurable with someone they were attracted to. Some of these initial relationships were abusive, leading to female respondents feeling that not being attracted to men was due to sexual trauma, when in fact they were only attracted to women. Participants also discussed feelings of shame, isolation, depression, and suicidal ideation due to their sexual orientation as they felt “other” and different.

Keiser et al. (2019) used a quantitative approach to measure the perceived inclusivity of sex education, with 263 participants recruited. The study sought to understand whether exclusive sex education affected the participants’ levels of anxiety and depression in adult life. The results suggest that sexual minority students who received decidedly heteronormative and exclusive sex education, demonstrate higher levels of anxiety, depression, and suicidal ideation.

The dominance of heterocentric RSE as normative is most evident in abstinence sex education. Hoefer and Hoefer (2017) reported on the impact of abstinence-based sex education in one state in Northern America whose participants *all* reported shame or guilt due to their sexual orientation. This resulted from teachers shutting down discussions of a sexual minority, labeling homosexual sex as dangerous, and promoting heterosexuality as the only option. This environment led sexual minority students to hide themselves in the sex education classroom and not ask the questions they needed to due to fear of discovery and harassment. Estes (2017) used qualitative interviews to discover the experiences of ten gay, lesbian, pansexual, or bisexual young adults aged 19 to 25 on the abstinence sex education classes received in a high school in Northern America and likewise found that students felt the sex education they received was irrelevant to them as it did not address the issues they wanted to understand.

Two papers pointed to the advantages of inclusive RSE in schools, which created an environment of more tolerance in general. Baams et al. (2017) focused their study on whether inclusive sex education reduces LGBTQ bullying in the school environment. They found that inclusive sex education led to a school environment that became more tolerant, with teachers and students more confident in intervening when witnessing homophobic abuse. Inclusive RSE placed greater emphasis on STI prevention for boys and external anatomy education for girls. Consequently, the school climate changed as teachers did not merely discuss heterosexual sex, pregnancy prevention, and marriage. However, Baams et al.'s (2017) study showed some variation according to gender. Girls showed more acceptance of different sexualities in their peer group and displayed the courage to challenge homophobic abuse more than their male counterparts, despite receiving the same inclusive curriculum.

Coll et al.’s (2018) study carried out in the UK used a youth participatory approach with 43 sary school-aged pupils over an 18-month period. The students as active researchers in the study. Through interactive group discussions, the young people discussed the unwritten rules in the school environment around gender and sexual orientation, noting that if a boy “hangs around” with a girl, he will be considered gay, and it would be considered unacceptable to demonstrate public affection with a member of the same sex. Sex education resources mirrored this environment, as one student noted: *“it (curriculum) presumes that all couples are straight”* (Coll et al., 2018, p. 166).

#### Theme 2: Poor Sexual Health Literacy Due to Disengagement

Feeling unsafe emotionally and physically was a common theme in LGBTQ participants who had experienced both homosexual and heterosexual relationships, and this appears to be as a direct result of receiving heteronormative sex education only. Formby and Donovan (2020) looked at young people's experiences of RSE and how they seek the information they have not acquired in class. Researchers conducted a questionnaire before using art and drama to prompt discussion of relationships and domestic abuse. A quarter of the participants had experienced domestic abuse, and all felt their education around same sex domestic abuse was non-existent, leaving them to develop their own understanding of healthy relationships through first partners or online information. This left them at heightened risk of abuse compared to their heterosexual peers, who had received generic information about male to female abusive relationships. The study also found that LGBTQ young people accessed to support and information from more experienced members of the LGBTQ community, which could be problematic depending on age differences and the potential power imbalances in these relationships. These findings were apparent in other studies reviewed (Estes, 2017; Gowen & Winges-Yanez, 2014; Grant & Nash, 2018; Hobaica & Kwon, 2017).

Grant and Nash (2018) discovered poor sexual health literacy led the women in the study to feel responsible for the abusive relationships, as it was inferred that it was their responsibility to regulate their partners’ behaviors. This could be said for many women experiencing domestic abuse in relationships, but is confounded further within homosexual relationships due to the absence of established norms within a healthy same-sex relationship (Grant & Nash, 2018). Formby and Donovan (2020) reiterate these findings, reporting that a quarter of the young people they surveyed knew of LGBTQ friends who had been in an abusive relationship. They felt this was due to the narrative of domestic abuse as a heterosexual problem, perpetrated only by men, leaving out descriptions of coercive control or emotional abuse. Participants in Gowen and Winges-Yanez’ (2014) study felt that discussions around relationships would help to maintain safe boundaries in relationships and therefore all forms of relationship should be covered.

Estes (2017) found that the participants who received abstinence-based sex education felt uninformed, mainly due to an overly biological approach to sex education, which explained how a baby is created and did not allow discussion around sex using contraception in any form. Gowen and Winges-Yanez (2014) found that bisexual and lesbian young women are at greater risk of STIs due to lack of knowledge around female contraception, with participants stating that they found out about STI transmission between women through trial and error and via supportive social media groups. Moreover, some women in this study perceived there to be no need to access cervical screening programs as they were not having penetrative sex with men. Hobaica and Kwon's (2017) study also confirmed findings of poor health literacy, which resulted in experiences of risky sexual practices, uncertainty about the level of protection required for homosexual sex, attempts to explore same-sex relationships through trial and error with first partners, leaving LGBTQ young people at risk of physical and emotional abuse. Lack of knowledge and confidence around the mechanisms of same-sex intercourse also resulted in homosexual young people engaging first in heterosexual relationships often while intoxicated with alcohol or drugs, leading to unsafe sex, feelings of shame, and poor mental health (Hobaica & Kwon, 2017).

#### Theme 3: Self-Taught RSE via Online Resources

Participants in four studies quoted the internet as a useful resource when attempting to navigate early homosexual relationships, with social media, charitable websites, and pornography being the main sources for education around the mechanics of same-sex relationships (Formby & Donovan, 2020; Gowen & Winges-Yanez, 2014; Grant & Nash, 2018; Hobaica & Kwon, 2017).

Sign posting to safe online resources was a key recommendation by Gowen and Winges-Yanez (2014) and Formby and Donovan (2020). Formby and Donovan's research found that LGBTQ young people access pornography to make up for the poor provision of RSE. LGBTQ young people were most likely watch pornography online by themselves as this enables young people to peruse information privately without fear of discovery. Online resources were overwhelmingly positive in helping young people to create their sexual identity (Formby & Donovan, 2020).

Grant and Nash (2018) discovered the lengths lesbian and bisexual young people go to in creating supportive social media networks to provide education to new members of their community. However, Hobaica and Kwon (2017) observed that participants in their study did note the limitations of online information, in particular pornography, as there was little evidence of contraception in pornographic films, especially between female partners. Moreover, information relayed by peers or sexual partners can be open to manipulation within abusive relationships, reinforcing the argument that both schools and parents have a responsibility to equip young people with the tools they need via face-to-face teaching, through the creation of inclusive environments and by sign posting to safe online resources (Formby & Donovan, 2020).

## Discussion

Overall, research examining the inclusivity of RSE in schools is still in its infancy. Medical models of intercourse and its risks dominate public sexual health discourses (Rabbitte, 2020). Evidence from our rapid review further highlights the consequences of non-inclusive sex education for LGBTQ young people, who are left ill-equipped to navigate their sexual health and relationships safely, resulting in mental health problems, abusive relationships, and potentially suicide (Metro, 2016; Stonewall, 2017).

The three themes extracted clearly demonstrate that to ensure LGBTQ young peoples’ emotional health and self-esteem is promoted and they can safely navigate sex without engaging in risky behaviors, the dominance of heteronormative RSE in UK schools needs to be reviewed. The example of sex abstinence programs in North America which can be promoted by individual state policies that prohibit the inclusion of sexual minority education by law (Hoefer & Hoefer, 2017) seemed to have been mirrored in the UK up until 2003. The consequences of this are still being felt by LGBTQ young people in the school environment today (Aranda et al., 2018; Stonewall, 2017) creating minority stress and subsequent mental health issues (Meyer, 2003). It must however be recognized that many teaching staff aims to provide leadership and a safe space for students to be able to speak about their sexual orientation (Preston, 2013). In the UK, Stonewall (2017) found that between 2007 and 2017, bullying of sexual minority and gender minority students had fallen by a third and schools were more likely to recognize homophobic abuse as a problem to be taken seriously and stopped. However, despite the changes in the RSE curriculum since 2003, a large UK study with 7,126 young people aged 16–25 revealed that LGBTQ young people felt that their time at school left them feeling isolated, unsupported, and frequently bullied (Metro, 2016). Lack of experience, knowledge, and role modeling means that there is a greater chance for LGBTQ young adults to get involved in abusive relationships. However, this is not always recognized nor reported. Research by Donovan and Barnes (2020) and Metro (2016) found that this was confounded by the LGBTQ community's lack of trust in statutory services, which resulted in a reduction in the reporting of abuse.

Adopting an inclusive RSE approach is not however a case of simply adding examples of same-sex intercourse. It is a much more nuanced subject. Research is needed to determine how best to approach same-sex intercourse to ensure that alienation does not result, especially for young males who experience stricter “peer-playground hierarchies” than females. Indeed, participants in Hobaica and Kwon's (2017) study questioned whether discussion of homosexuality in school sex education would create a base from which homophobic abuse could occur. To fully engage male students, toxic masculinity must be taken into account. Hilton's (2007) study of 16–17-year-old boys in the UK demonstrated that young men wanted safe, non-critical spaces to discuss sexuality, reflecting a desire to tackle gender norms, but only with support and protection from name-calling and the possibility of peer rejection (Hilton, 2007).

As Baams et al. (2017) found, there may be the need to distinguish between the types of education offered with a mixture of single-gender group conversations, mixed-gender group conversations, and opportunities for one-to-one conversations with trusted and trained professionals such as a school nurse or RSE teacher champions.

The school level (primary or secondary) is also important to consider, both of which imply an environment traditionally constrained by the national curriculum and the values and principles of parents and governors. Higher education institutions, such as universities, can be seen as more inclusive with much less homophobic discrimination being reported (Ellis, 2008; Stonewall, 2018). Hobaica and Kwon (2017) noted in their study, which only featured college-educated students, that some young people changed their sexual identity upon learning more about sexual orientation when leaving high school and entering college.

In an attempt to discover more information, young people turn to online resources which bring risks in-itself as these sources can be misinformed and pose potential threats from sexual predators. In this respect, it is even more important that schools equip young people with the skills and knowledge on how to navigate online information while providing a list of trusted sources. However, effective signposting does not appear to be happening within UK schools. Young people overwhelmingly report a lack of posters or leaflets around their school on LGBTQ issues and how to access contraceptive advice (Metro, 2016). When young people access pornography online, Horvath et al. (2013) found adolescents experienced greater confusion about their values and were more sexually uncertain the more pornography they viewed. One survey found that over half of young people had not been actively searching for pornography online, but had been sent it by someone else or were directed to it via an internet “pop-up” (Martellozzo et al., 2020). According to Martellozzo's survey, 42% of young people reported watching pornography gave them ideas for “things they wanted to try out,” subjects identified by LGBTQ young people as missing from their heterocentric sex education sessions. Notwithstanding, a study by Robinson et al. (2014) found that participants who only accessed online RSE information felt more isolated than participants who received a combination of online and face-to-face education, again highlighting the importance of classroom-based RSE as it provides the opportunity for dialog and exchange of experiences.

All young people are open to online risks, but homosexual young people are more likely to take risks in their sexual lives, and so may need more protection from these harms. Hobaica and Kwon (2017) found the majority of LGBTQ young people they surveyed used online resources extensively as they were seen as essential to their sexuality education. Searching for information on safe sex practices, sexual positions and relationships have both positive and negative outcomes.

All young people benefit from inclusive sex education, as it is recognized that sexual identity can change (Abbott et al., 2015) and a variety of sexual practices are common in both heterosexual and homosexual relationships, leaving all young people in need of the knowledge to protect themselves during anal and oral sex (Carpenter, 2001). Young people would benefit from sex education that does not merely focus on the health risks of being LGBTQ (Gahagan & Colpitts, 2017), but provides them with the education to prevent, recover from and resolve the sex and relationship challenges they are likely to experience throughout their lifetimes (Carver, 1998). It is further hoped that with continued support from school nurses and the new mandatory relationships education, school staff will receive up-to-date inclusive RSE training. Historically, teachers have viewed homosexual pupils within their own ideas of gendered stereotypes. Many have not recognized the impact of stress on minority students, perceiving that there was little direct bullying or physical abuse of homosexual pupils and assuming that they were a tolerant school (Preston, 2013).

### Limitations

The review focused only on publications in the English language, in comparable education systems. The inclusion of gray literature and conference proceedings was beyond the scope of the rapid review. The quality of the research included in the review was good to medium and only one study provided a longitudinal perspective (Baams et al., 2017). Most studies were limited in sample size, highlighting the need for research into a wider demographic of young people in the LGBTQ population, including young people of color and students at all levels of education and establishments.

## Conclusion

The findings from this review clearly indicate that young people do not want their educators to assume they are heterosexual and need a broad range of knowledge, resources, and skills to safely navigate their future relationships. In order to ensure RSE is inclusive and relevant to all, policy must incorporate mandated, interactive training for all teachers and should include healthy behaviors in same-sex relationships, contraceptive advice for all types of relationships, and a whole school culture, which incorporates discussion of same-sex relationships within all subjects of the curriculum. This is imperative as increasing knowledge of same-sex relationships creates a safer environment for all young people to explore their identity and sexuality without fear or discrimination and will encourage students and school staff to challenge homophobic abuse in the school environment (Baams et al., 2017). Local school public health and other charitable services are well placed to work collaboratively to provide inclusive online RSE material for young people to access and schools must ensure all young people are regularly signposted to these resources. Young people should be consulted on their views of what to include and how to deliver sexuality education at school to ensure a fully inclusive curriculum (Estes, 2017; Gowen & Winges-Yanez, 2014; Grant & Nash, 2018; Hobaica & Kwon, 2017; Hoefer & Hoefer, 2017). Regular research at a local level through youth participatory research methods (Coll et al., 2018) and student evaluation should help educators keep up with their students, hear their views, and adapt their teaching to the topics young people feel they need to know within their generation's cultural climate.
